# Physiological and Molecular Responses to Main Environmental Stressors of Microalgae and Bacteria in Polar Marine Environments

**DOI:** 10.3390/microorganisms8121957

**Published:** 2020-12-09

**Authors:** Chiara Lauritano, Carmen Rizzo, Angelina Lo Giudice, Maria Saggiomo

**Affiliations:** 1 Marine Biotechnology Department, Stazione Zoologica Anton Dohrn, Villa Comunale, 80121 Napoli, Italy; 2 Marine Biotechnology Department, Stazione Zoologica Anton Dohrn, Villa Pace, Contrada Porticatello 29, 98167 Messina, Italy; carmen.rizzo@szn.it; 3 Institute of Polar Sciences, National Research Council (CNR-ISP), Spianata S. Raineri 86, 98122 Messina, Italy; angelina.logiudice@cnr.it; 4 Research Infrastructure for Marine Biological Resources Department, Stazione Zoologica Anton Dohrn, Villa Comunale, 80121 Napoli, Italy; maria.saggiomo@szn.it

**Keywords:** polar environments, microalgae, bacteria, stressors, stress responses, -omics analyses

## Abstract

The Arctic and Antarctic regions constitute 14% of the total biosphere. Although they differ in their physiographic characteristics, both are strongly affected by snow and ice cover changes, extreme photoperiods and low temperatures, and are still largely unexplored compared to more accessible sites. This review focuses on microalgae and bacteria from polar marine environments and, in particular, on their physiological and molecular responses to harsh environmental conditions. The data reported in this manuscript show that exposure to cold, increase in CO_2_ concentration and salinity, high/low light, and/or combination of stressors induce variations in species abundance and distribution for both polar bacteria and microalgae, as well as changes in growth rate and increase in cryoprotective compounds. The use of -omics techniques also allowed to identify specific gene losses and gains which could have contributed to polar environmental adaptation, and metabolic shifts, especially related to lipid metabolism and defence systems, such as the up-regulation of ice binding proteins, chaperones and antioxidant enzymes. However, this review also provides evidence that -omics resources for polar species are still few and several sequences still have unknown functions, highlighting the need to further explore polar environments, the biology and ecology of the inhabiting bacteria and microalgae, and their interactions.

## 1. Introduction

Oceans cover more than 70% of the Earth’s surface, and host diversified habitats and adapted organisms. Aquatic ecosystems are dominated by different dynamics in which phyto- and bacterioplankton take on crucial roles. In the last decade, aspects concerning their mutual interactions and their influence on a global scale have gained scientific interest for ecological and, more recently, biotechnological insights. In addition, recent advances in -omics technologies, such as genomics, transcriptomics, proteomics, and metabolomics, applied to microalgae and bacteria have shed light on biosynthetic pathways that can be involved in the mechanisms of adaptation to different environments and production of defense metabolites with possible industrial applications [1,2,3,4,5,6,7,8,9,10,11].

Phytoplanktonic organisms are responsible for almost 50% of global photosynthesis, thus regulating carbon and oxygen fluxes at a globally scale [12]. On the other hand, heterotrophic bacteria cover about a quarter of the total biomass in aquatic environments in terms of abundance [13]. Environmental parameters can affect microalgae and bacteria in different ways, because of their different needs for proliferation and growth. Microalgae, for example, need a specific light regime to carry out their physiological processes, which most bacteria do not require, so they are particularly sensitive to the combination of low temperature and seasonality of solar irradiance [14]. Changes in light conditions can therefore be considered a direct determinant for the distribution and adaptation of microalgae, while they only have indirect effects for bacteria. Differently, other factors such as temperature, salinity or pH affect both microalgae and bacteria, affecting their distribution in the variety of microhabitats occurring in polar areas. If microalgae are found mainly distributed in habitats with specific light irradiance differences (e.g., seawater, sea-ice, snow and rock surfaces), bacteria are also well established in other environmental matrices, such as sediments, soils and atmosphere, thus having to cope with different pressures.

Taken separately, phytoplankton and bacterioplankton numerically dominate aquatic environments [15], and possess roles at the respective cornerstones of ecosystem functioning, being the main architects of primary production and decomposition processes, respectively. However, it is thanks to poorly investigated direct or indirect interactions that are responsible for their main ecological and large-scale effects [16]. The relationships occurring between phytoplankton and heterotrophic bacteria are very intimate, spanning from cooperation (mutualism) to competition (parasitism), but such interactions have been poorly studied due to the difficulty to separate them from one another.

The ecological coupling between phytoplankton and bacteria is often based on a reciprocal exploitation of resources such that each species is a consumer of a resource provided by the other. Bacteria fulfill their carbon needs by assimilating the fraction of dissolved organic carbon (DOC) supplied by phytoplankton cells and the complex products deriving from algal biosynthetic processes, or from dead phytoplankton biomass [17]. On the other hand, phytoplankton can benefit from the production of special molecules (i.e., siderophores), vitamins or micronutrients supplied by bacteria, as well as from the limiting macronutrients made available by bacterial remineralization processes [18,19]. The bacterial component is therefore to be seen in a different light, which goes beyond the simple role of decomposers, by assuming a key role in the promotion of algal growth. Being so intimately linked, environmental stressors that affect directly one of these two components could therefore indirectly influence the other.

**Figure 1:**
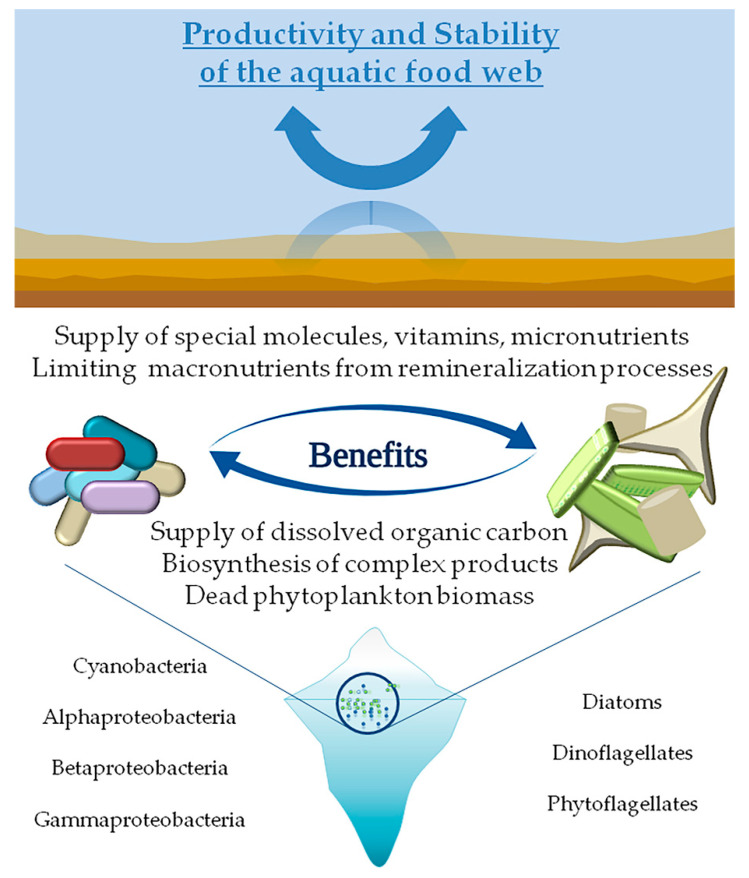
Schematic view of the ecological coupling between phytoplankton-bacteria with benefits deriving from reciprocal exploitation of resources.

A schematic view of the interaction between bacteria and microalgae is provided in Figure 1. In polar environments such relationships are expected to be even more sensitive and with peculiar characteristics, since harsher environmental conditions require more complex adaptation and mechanisms of interaction. Arctic and Antarctic marine ecosystems are characterized by a high level of biological and genetic diversity and are still largely unexplored compared to more accessible sites. This review aims to highlight some of the main survival strategies phytoplankton and bacteria have adopted to cope with extreme cold conditions through the application of -omics technologies.

### Environmental Factors Driving Microbial Life in Cold Marine Environments

Arctic and Antarctic regions are similar in that they are strongly affected by snow and ice cover changes and by extreme photoperiods (with periods of total darkness) and low temperatures. These regions constitute 14% of the total biosphere, including cold habitats such as sea-ice, permafrost and cold soils, glaciers, lakes and subterranean water bodies. A number of environmental factors can characterize, either singly or in combination with one another, terrestrial and marine low-temperature environments [20] with repercussions on living organisms at different trophic levels. As an example, since phytoplankton are highly dependent on light availability, primary production is highly pulsed and primary consumers store carbon as lipids during the summer to survive during winter.

Arctic marine ecosystems cover an area of approx. 20 million km^2^. Permanent sea-ice cover annually represents 40%, but can reach 75% at the winter maximum, resulting in a wide seasonal variation in solar radiation and air temperatures. Over the extensive continental shelf areas, surface seawater temperature varies from <0 in winter to +5–10 °C during summer [21].

The Southern Ocean represents 9.6% (approx. 35 million km^2^) of the world’s oceans, and is predominantly very deep (about 4000 m). About 21 million km^2^ are ice-covered at the winter maximum, but only 7 million of this cover persists at the summer minimum. Antarctic surface seawater temperatures annually range between −1.86 °C and +0.3 °C. The Western Antarctic Peninsula has experienced one of the strongest warming events in the world [22]. In addition to reduced sea-ice extent, 80% of the glaciers on the Antarctic Peninsula are retreating as well. Changes in sea ice and in glacial melt have an impact also on salinity and can affect phytoplankton community composition [23]. The annual retreat and melting of sea ice in the austral spring stratifies the upper ocean, triggering large phytoplankton blooms [24]. The magnitude of the blooms is proportional to the winter extent of ice cover, which can act as a barrier to wind mixing. The Western Antarctic Peninsula region has warmed by 7 °C over the past 50 years, and sea ice duration has declined by almost 100 days since 1978, causing alterations in phytoplankton productivity in the Northern peninsular region [25] with implications for higher trophic levels [24].

In common with the Arctic, but distinct from other coastal ecosystems, the variations in sea ice distribution and the freshwater inputs from melting sea ice and glacial ice are the dominant influence on ecological and biogeochemical processes in Antarctic coastal systems. In contrast to the Arctic, in Antarctica freshwater input derives exclusively from melting ice (e.g., pack ice and icebergs) or precipitation as there are no major rivers discharging into the Southern Ocean. Another difference between the Arctic and Antarctic pelagic ecosystems is the change in light and in surface sea-ice that occurs at a seasonal and annual scale, respectively.

Foremost, sea-ice represents a critical community-structuring factor in polar marine systems. Sea ice duration, extent and seasonality are the principal physical determinants of variability in ecosystem dynamics in coastal Antarctic marine ecosystems. Its presence impedes surface seawater mixing, affects freshwater and heat fluxes, and in combination with snow cover reduces the availability of light for primary production. Seasonal or annual freezing-melting cycles of large amounts of seawater lead to changes in physical and biogeochemical properties of both surface ocean and sea-ice [26]. During sea-ice growth, salt ions are concentrated as brines, which are partially retained within the ice in brine channels and pockets and partly ejected into the upper ocean. This latter ice-ocean interaction process leads to surface layer modification in density, as well as to the induction of thermohaline convection. Microorganisms colonizing sea-ice experience temperature-driven fluctuations in salinity (from 0 to over 200), with subsequent possible osmotic stress. In turn, variations in irradiance quantity and quality, increased temperature and nutrient supply, and lowered salinity occur in the upper ocean during ice melting [27].

The physical environment shapes microbial communities through evolutionary adaptation and acclimation responses to long- and short-term changes. Rising temperatures and freezing-thawing phenomena (with their consequences) are among the main environmental stressors acting on the microbial life in a global change scenario. In the following paragraphs we discuss the physiological and molecular responses of microalgae and bacteria to environmental constraints in the Arctic and Antarctic marine environments.

## 2. Microalgae

Marine algae thrive in polar regions, forming rich communities dominated mainly by brown algae [28,29]. Benthic microalgae are well represented and may account for around 40–50% of the primary production in polar waters [30]. Algal cell concentrations in sea-ice vary by up to six orders of magnitude and algal production rates show similar variation [31]. Biomass accumulation and production depend on the vertical position of sea-ice algae in the ice cover, and are controlled by various environmental parameters like salinity, light, nutrients, temperature and pH (e.g., [32]). Due to different oceanographic and geological characteristics, microalgal communities of Antarctica and the Arctic strongly differ. Antarctica is characterized by a high endemism, whereas in the Arctic only a few endemic species occur. In contrast to the Antarctic region, where micronutrient levels never limit algal growth, nutrient levels in the Arctic regions are depleted during the summer season [33]. Both regions have a strongly seasonal changing light regime, due to ice covering throughout the winter months. Diatoms comprise from 40 to 60% of the sediment in the Antarctic region [34]. During the spring and summer seasons, the haptophyte *Phaeocystis antarctica* is the dominant taxa within the phytoplankton community [35,36] contributing with about 75% of the primary production in the Southern Ocean, supporting most food webs in Antarctica [37]. In the Arctic Ocean, total freshwater inflow increased by 7% from 1936 to 1999 [38], with potential for further increases with Arctic warming [39]. Increased freshwater discharge (resulting from climatic change) acts on biological communities, nutrient supply, turbidity, and the inflow of low salinity water [40]. Judging from sea-ice satellite data, sea-ice has declined by 17% at the end of the Arctic summer over the last 25 years [41]. Not only does it determine the physical properties of the underlying water column [42], it also incorporates a complex structure that is host to diverse algal communities, which grow in, on and under the ice. Here, the algae grow within narrow channels that originate during ice formation. The salinity within these channels is a function of the average surface temperature and can be more than double that of the surrounding seawater [43]. However, in summer, when ice begins to melt, the salinity of the melted sea-ice can be as low as one-third that of normal seawater and may influence the growth rates of ice algae [44]. The fast-ice algal community in all areas is usually dominated in the bottom ice by the diatoms *Entomeneis kjellmannii*, *Nitzschia stellata*, *Thalassiosira australis*, *Berkelaya adeliense* and *Navicula glaciei*, although many other taxa have also been recorded [37]. Melting of massive snow/ice accumulations and consequent run-off have strongly impacted the physical and biological processes of near-shore pelagic and benthic communities [45]. Table 1 summarizes the main physiological and molecular effects/responses observed for microalgae exposed to common polar environmental stressful conditions, as discussed in the following sections.

### 2.1. Salinity

Marine phytoplankton species show broad salinity tolerance with many species capable of growing within a wide salinity range. Coastal species are usually more euryhaline compared to oceanic species, because they are less subject to fluctuations. While ice algae can still be physiologically active at salinities as high as 100‰, most show a severe inhibitory response to photosynthesis at salinities greater than 60‰ [67]. A reduction in salinity has been shown to suppress photosynthetic capacity and efficiency in Arctic algae. It would seem that most sea-ice algae are more tolerant to decreased salinities than increased salinities [68]. Microalgae are vulnerable to high salinity because they have to grapple not only with ionic imbalance and osmotic stress but also with the generation of reactive oxygen species (ROS) interfering with photosynthesis. Salt tolerant marine strains are valuable models to identify and characterize genes associated with salinity resistance.

Some polar marine diatoms (*Nitzschia lecointeii*) change in biochemical composition (carbohydrate, protein, fatty acid, particulate organic carbon and nitrogen content) in response to salinity changes, even when growth rate or photosynthesis was unaffected [44]. *Fragilariopsis cylindrus* accumulates proline in excess, when grown at salinity 70‰. The solutes can also be released rapidly from marine algal and bacterial cells into the environment in response to relatively large salinity downshifts (>14‰) [69]. Once in the surrounding environment, microalgal-produced compatible solutes can be taken up by other members of the microbial community, such as heterotrophic bacteria, for the purpose of osmoregulation or as a source of carbon, nitrogen or energy [46]. This catabolism of compatible solutes by bacteria can lead to the production of climate-active gases such as dimethylsulfide (DMS) from dimethylsulfoniopropionate (DMSP) [47]. In *Chlamydomonas* species, stress caused by high salt concentration slows down cell division, growth rate, reduces size, ceases motility, and triggers the so-called “palmelloid” formation. “Palmelloid” is a temporary stage created when *Chlamydomonas* cells are exposed to unfavorable conditions and is characterized by loss of flagella, minimum of two cells clustering, increased secretion of exopolysaccharide (EPS), cells surrounded by an EPS matrix sharing a common membrane, and cell wall thickening [48].

Regarding other green algae, cells with rigid cell walls like *Chlorella* have limited ability to change cell volume, while *Dunaliella* lacks a rigid cell wall and can rapidly change the volume during high salinity stress. The limited ability to change cell volume depends on organic solutes for osmoregulation, such as glycerol and proline. Glycerol is produced by most salt sensitive algal species under high saline stress. It is an end-product metabolite and thus production and accumulation does not interfere with other metabolic pathways [70]. Proline is another osmoregulatory solute that increases with increasing salinity [48]. The transport of ions through the cell membrane is another important strategy to cope with high salt stress by maintaining the intracellular ion balance. According to the same studies, high concentrations of Na^+^ can interfere with the uptake of other cations, especially K^+^. Since K^+^ have a physiological function in algae, it is necessary to maintain the cytosolic K^+^/Na^+^ ratio under high saline conditions. Up-regulation of membrane transport proteins can confer tolerance to high salinity in halotolerant algae species by actively transporting K^+^ ions through membrane transport proteins. In addition, most algal cells show a characteristic accumulation of lipids under salt stress. Lipids act as storage reserves for salt stressed cells and can be immediately degraded when stressed cells are introduced to optimal conditions [48].

### 2.2. Light

Photoautotrophic microorganisms [71] use solar radiation as a principal energy source to drive physiological processes such as photosynthesis and growth. However, high levels of solar ultraviolet radiation (UVR), and especially UV-B, are considered to be a stressor for many physiological processes and will cause damage to DNA [72], inhibit photosynthetic rate and inactivate enzymes [51]. These biologically harmful effects of UVR can negatively affect the diversity and species richness of algal communities [73]. Goes et al. [52] found that the main effect of elevated UVB levels on fatty acid synthesis of Antarctic phytoplankton was an increasing concentration of saturated fatty acids, and a decreasing concentration of polyunsaturated fatty acids. The desaturation process is dependent on the production of ATP and the uptake of inorganic nitrogen, both of which are affected by increased levels of UVB [52]. Because UVB affects the uptake of nitrogen, the biochemical changes can look similar to those caused by nitrogen limitation. There is also a small increase in the proportion of C18 polyunsaturated fatty acids (PUFAs) with distance above the ice/water interface. Light attenuation by sea-ice might protect primary producers from UVR, but could also limit primary production, and consequently many ecosystem processes are closely tied to the few months of open water [74].

### 2.3. Nutrient

The major part of the Southern Ocean is considered a high-nutrient and low-chlorophyll (HNLC) area, primarily due to the limitation of micronutrients, such as iron (Fe) [54]. Nevertheless, phytoplankton may bloom if Fe is available and if light and mixing conditions are suitable. The size of the specific composition of microalgae and in particular of diatoms is influenced by the presence of Fe [53]. However, Fe is not the only factor that controls phytoplankton blooms [75]. Phytoplankton cells during the austral summer produce photo-protective pigments in order to protect their photosystems under high irradiance [49,50]. As a result, algae reduce the abundance of Fe cellular components (e.g., cytochrome b6f complex), lowering the electron transport capacity and decreasing the light harvesting pigments by converting them into protective ones [76]. These combined effects lead to a decrease in growth rates caused by a reduction in both nitrogen fixing capacity and photosynthetic protein production. Therefore, the distribution of phytoplankton in the Antarctic ocean is mainly influenced by Fe and light [53].

### 2.4. pH

Current levels of atmospheric CO_2_ are predicted to be more than double by 2100 (Intergovernmental Panel on Climate Change IPCC 2007) and this will be accompanied by a sharp decline in pH. Ocean acidification (OA) is a direct ocean response to increased anthropogenic CO_2_ emissions. Ocean acidification is occurring in all surface waters but the strongest effects will be experienced in polar ecosystems with significant effects on all trophic levels.

Polar waters are particularly vulnerable to ocean acidification due to the increased solubility of CO_2_ [77]. Polar oceans are chemically sensitive to anthropogenic acidification due to their relatively low alkalinity and correspondingly weak carbonate buffering capacity. The Arctic experiences greater seasonal warming, and lower summer pH (8.15 vs. 8.5), than the Antarctic. Despite a larger uptake of inorganic carbon by summer photosynthesis, the Arctic carbon system exhibits smaller seasonal changes than the more alkaline Antarctic system. In addition, excess surface nutrients in the Antarctic may allow mitigation of acidification, via CO_2_ removal by enhanced summer production driven by iron inputs from glacial and sea-ice melting. These differences suggest that the Arctic system is more vulnerable to anthropogenic change due to lower alkalinity, enhanced warming, and nutrient limitation [78].

Studies have shown contrasting results on the effects of ocean acidification on microalgae. Predictions range from ocean acidification favoring larger diatoms to early senescence in some algal species and possible disappearance in some areas of the oceans [55]. Although many species, including most diatoms, possess effective carbon-concentrating mechanisms (CCM), they may still benefit from decreased costs for carbon acquisition [79]. The direct effect of oceanic pH on microalgal cells is less clear but recent studies have demonstrated that it can modify intracellular pH and affect membrane potential, energy partitioning and enzyme activity [56]. Furthermore, microalgae living in extreme environments have been reported to tolerate short term exposure over a very large range in pH [77,80].

### 2.5. Temperature

Polar microalgae have a great ability to persist at low temperatures. In recent decades, global warming has been associated with rising temperatures and carbon dioxide (CO_2_) levels, sea level rise, iceberg thinning, and sea-ice retreats [81]. In the last 30 years, in the Antarctic Peninsula, during the austral summer, there has been a 12% decrease in the abundance of phytoplankton [82]. Temperature influences many microalgal physiological processes such as biochemical composition [83], photosynthesis [84] and gene expression [85]. One of the main effects of heat stress is on lipids and fatty acids, as these are the energy source for stress adaptation in algae [86].

In relation to cold adaptation, polar algae have similar metabolic strategies including (a) maintenance of membrane fluidity thanks to unsaturated fatty acids that prevent rigidity of membrane lipids; (b) maintenance of sufficient rates of enzyme-catalyzed reactions for key metabolic processes; (c) evolution of cold shock and antifreeze proteins, and (d) adaptations of the photosynthetic electron transport chain to function at cold temperatures (reviewed by [57]). These physiological characteristics allow Antarctic and Arctic algae to complete their life cycles at low temperatures. On the contrary, differential gene expression analyses in temperature increase experiments for the Antarctic alga *Chlorella* UMACC 234 showed differentially expressed genes (DEG) mainly associated with photosynthesis, carbohydrate metabolism, electron transfer, and cell maintenance. (e.g., Photosystem II P680 chlorophyll a apoprotein CP47, aldose 1-epimerase, a putative oxidoreductase) [85].

### 2.6. -Omics Studies on Microalgae from Cold Environments

The recent advancement of new technologies such as -omics technologies has led to a better understanding of the physiology of marine microalgae and their adaptation mechanisms to extreme polar environments. In recent years, various studies on polar microalgal genomes, transcriptomes, proteomes and metabolomes have been performed, as well as studies on expressed sequence tag (EST) libraries and single gene identification. Genomes of microalgae from polar environments are available for very few species. The first eukaryotic microorganism from a polar environment to have its genome sequenced was the chlorophyte *Coccomyxa subellipsoidea* C-169 [87]. In its 48.8 Mb genome, Blanc and co-workers found several protein families over-represented, such as proteins involved in lipid metabolism, transporters, cellulose synthases and short alcohol dehydrogenases. In particular, fatty acid synthases, elongases, lipases and desaturases were among the most enriched gene families, suggesting the importance of lipid metabolism for cellular homeostasis maintenance (e.g., for membrane fluidity). On the contrary, the genome lacked components of the glycosyl phosphatidyl inositol anchoring system, pyruvate phosphate dikinase and the photosystem 1 reaction center subunit N, which were found for other sequenced chlorophytes (*Chlorella variabilis* and *Chlamydomonas reinhardtii* from temperate environments). The authors suggested that some of these gene losses and gains could have contributed to polar environment adaptation.

Recently, the genome of the cold-adapted diatom *Fragilariopsis cylindrus* has been sequenced [58]. Authors compared its genome with temperate diatoms. They found that about 24.7% of the *F. cylindrus* genome had genetic loci with highly divergent alleles which were also differentially expressed across various environmental conditions, such as darkness, low iron, freezing, elevated temperature and increased CO_2_. Divergent alleles were suggested to be involved in *F. cylindrus* adaptation to the Southern Ocean environment giving new insights on genotypes impacting phenotypes and their adaptation to extreme environments. Of the genes supposedly involved in that adaptation were ice-binding proteins (IBPs), proton-pumping proteorhodopsins and chlorophyll a/c light-harvesting complex (LHC) proteins of which there was an unusually large number, probably involved in light stress response strategies.

EST library generation and array analysis approaches were also used to study polar microalgae. Mock and Valentin [59] performed an experiment on the psychrophilic diatom *F. cylindrus* under freezing temperatures. Gene expression analyses (macroarray) during a temperature shift from +5 °C to −1.8 °C was carried out under 3 and 35 μmol photons m^−2^ s^−1^. At 35 μmol photons m^−2^ s^−1^, genes encoding proteins of PSII (*psbA*, *psbC*) and for carbon fixation (*rbcL*) were down-regulated, whereas genes encoding chaperones (*hsp70*) and genes for plastid protein synthesis and turnover (elongation factor *EfTs*, ribosomal protein *rpS4*, *ftsH* protease) were up-regulated. In contrast, cold exposure at 3 μmol photons·m^−2^·s^−1^ induced down-regulation of *psbA*, *psbC*, and *rbcL*, but no increase in *hsp70*, *EfTs*, *rpS4*, and the *ftsH* protease, suggesting that these genes are probably involved only in cold shock response at higher light intensities. Mock et al. [88] also analyzed expressed sequence tags (ESTs) generated from *F. cylindrus* RNA isolated 5 days after *F. cylindrus* cells were shifted from approximately +5 °C to −1.8 °C. Most sequences were similar to genes coding proteins involved in translation, ribosomal structure, biogenesis, amino acid transport and metabolism, and post-translational modifications. Authors also highlighted the presence of sequences with still unknown functions that may be involved in cold adaptation and require further studies, especially because there are still few protist sequences available for comparison. An EST approach was also used to find candidate genes involved in acclimation to salt stress in *F. cylindrus* because the species was found to grow at extremely low temperatures and high salinities [60]. From 2880 cDNA clones, 1691 high-quality tentative unique sequences were assembled and 44.2% of these corresponded to sequences encoding different ionic transporters and antiporters, reflecting the requirement to re-establish the ion homeostasis disturbed by exogenous salt stress. In addition, several genes coding heat shock proteins (hsps), genes related to oxidative stress, and three key genes involved in proline synthesis were identified as well. Finally, four sequences showing significant similarities to ice-binding proteins (IBPs) were identified, giving new insights into their possible roles in salt-stress acclimation.

Hwang and co-workers [63] used a customized complementary DNA (cDNA) chip analysis to study relative expression profiling of 1439 ESTs of the diatom *Chaetoceros neogracile* exposed to temperature shift from 4 to 10 °C. 21.5% of probes were differentially regulated by thermal stress within three days. Cytoprotective genes were up-regulated, while photosynthetic genes were down-regulated. Also for this species, several sequences had unknown function or no similarity to known genes. Unexpectedly, a small set of genes encoding fucoxanthin chlorophyll a/c-binding proteins were up-regulated by the temperature upshift, suggesting different roles other than light harvesting. High irradiance was also tested by Park et al. [64] for *C. neogracile* identifying 577 differentially regulated transcripts out of 1439 (by using a custom cDNA chip during 6 h of treatment). As for *F. cylindrus*, transcripts related to photosynthesis were affected. Also the antifreeze protein gene (Cn-AFP) from *C. neogracile* was cloned and characterized [65]. Both the Cn-AFP coding region with and without the signal sequence were cloned, expressed in Escherichia coli and shown to have antifreeze properties (by measuring the thermal hysteresis and modified morphology of single ice crystals).

Recently, a transcriptome search analysis for DEAD-box RNA helicase gene family, known to have a possible role in the adaptation/response to stress conditions (e.g., cold adaptation) in other species, was performed for the Antarctic green alga *Chlamydomonas* sp. ICE-L for the first time [61]. Thirty-nine DEAD-box RNA helicase genes had been identified. Expression of various DEAD-box RNA helicase genes, such as *CiRH5*, *CiRH25*, *CiRH28*, and *CiRH55*, were found significantly up-regulated under freezing treatment, suggesting their possible role in freezing acclimation. A transcriptome analysis of the Antarctic green alga *Chlamydomonas* sp. ICE-L, isolated from sea-ice, was also performed in order to study freezing stress responding genes [62]. This approach indicated that many genes encoding PUFA synthesis enzymes, molecular chaperones and cell membrane transport proteins had high similarity to the genes from Antarctic bacteria, probably acquired through horizontal gene transfer from symbiotic microbes. In addition, a comparison with *Chlamydomonas reinhardtii* and *Volvox carteri* showed that various genes related to post-transcriptional, post-translational modification, and signal-transduction KEGG pathways, were absent indicating possible enhanced stress adaptation abilities of *Chlamydomonas* sp. ICE-L in a very cold habitat. In addition, differential expression analysis showed that the genes related to post-translational modification, lipid metabolism, and nitrogen metabolism were responding to the freezing treatment. In another transcriptome study of *C. reinhardtii* exposed to salt stress, a significantly increased expression of genes participating in the metabolism of carbohydrates, such as starch, sucrose, soluble sugar, and glucose was observed. Glycolysis is considered to play an important role in plant development and adaptation to multiple abiotic stresses, such as cold, salt, and drought. Results in fact showed an up-regulation of 31 genes involved in glycolytic processes [66].

Very recently, the transcriptome of the Arctic green alga *Chloromonas* sp. KNF0032 identified six IBPs, homologous with the previously reported IBPs from Antarctic snow alga *Chlamydomonas* sp. CCMP681 and Antarctic *Chloromonas* sp. These proteins were highly expressed at low temperature and had multiple exon/intron structures and low-temperature-responsive cis-elements in their promoter [89]. Three IBP proteins were also tested in vitro and further studied by using a transgenic plant system. Structural protein model prediction showed a β-solenoid form with parallel β-sheets and repeated TXT motifs, similar to the AidA domain-containing adhesin-like proteins. Altogether, data suggested that the IBP are a type of modified adhesins.

Finally, a recent metabolomics study focused on red and green snow algae communities from four locations in Ryder Bay (Antarctic Peninsula). Results showed that green communities (mainly consisting of *Chloromonas*, *Chlamydomonas* and *Chlorella*, bacteria, protists and fungi) were rich in proteins, chlorophyll and metabolites associated with nitrogen and amino acid metabolism. On the contrary, red communities (mainly consisting of *Chloromonas*, bacteria, protists and fungi) were characterized by higher carotenoid content and metabolites associated with carbohydrate and fatty acid metabolism. Altogether the research on polar microalgae has identified a series of peculiarities and genes that can be involved in the maintenance of cellular homeostasis and algal adaptation to freezing environments.

## 3. Bacteria

Morita [90] first distinguished cold-adapted bacteria in psychrophiles (a.k.a. cold-loving bacteria) and psychrotrophs (a.k.a. psychrotolerant or cold-tolerant bacteria). From a physiological point of view, psychrophiles cannot grow above 20 °C (optimal growth temperature of 15 °C or below). Conversely, psychrotrophs show a wider temperature range for growth than psychrophiles and fastest growth rates above 20 °C. As a consequence, these latter bacteria can better tolerate thermal fluctuations (e.g., diurnal or seasonal) than true psychrophiles that are more heat-sensitive and generally occur in permanently cold habitats. Moreover, the terms eury- and stenopsychrophile (indicating bacteria tolerating wide and narrow temperature ranges, respectively) can be used to refer to facultative psychrophiles/psychrotrophs and true/obligate psychrophiles, respectively [91,92]. The term “cold-adapted bacteria” will be preferentially used throughout this paper.

The bacterial communities from oceans of the two polar environments share about 15% of microbial species, denoting a markedly different bacterial composition due to the diverse environmental drivers occurring in Arctic and Antarctic habitats [93]. A fair level of endemism was demonstrated for Antarctic bacterial communities by the Tara Oceans Project [94], even if a similarity with temperate or tropical oceans was also evidenced. Among bacterial groups, *Alphaproteobacteria* (with a strong representation of SAR11 clade, due to the high plasticity of its members), *Flavobacteria* and *Gammaproteobacteria*, were the most abundant, while *Actinobacteria*, *Epsilonproteobacteria* and Firmicutes were reported as less abundant in both polar environments [95]. Some taxa seemed to be particularly influenced by seasonal variations, as in the case of bacterial clades within *Rhodobacteraceae*, uncultivated *Gammaproteobacteria* and *Bacteroidetes*, for which the main fluctuations have been recorded during summer and winter in different Antarctic areas [96]. *Flavobacteria* members were reported as dominant during algal blooms [96,97,98]. In Arctic waters, mostly affected by large riverine inputs, *Alphaproteobacteria* (including the SAR11 clade), *Flavobacteria* and *Gammaproteobacteria* members dominate [99]. While it has been suggested that *Gammaproteobacteria* occur primarily near coastal areas [100,101], *Alphaproteobacteria* are more represented far from coastal areas [99,102,103], and are scarce in the ice. By contrast, several authors [104,105] showed that *Flavobacteria* are present to a higher extent in the Arctic ice. A strong terrestrial influence was evidenced in Arctic samples [106], while seasonality seems to affect both areas, with few taxonomic groups detected during winter and autumn surveys [107]. Brinkmeyer et al. [106] evidenced a strong overlap in the sea-ice bacterial community composition in both poles, with the dominance of Proteobacteria and CF group members.

Interestingly, as discussed below, the recruitment of bacteria into sea-ice is correlated to the presence of particles or microalgae, suggesting attachment to their surfaces [108]. The main driving factor for bacterial communities in sea-ice is temperature that is probably correlated with phytoplankton blooms due to increasing temperatures and the consequent invasion of open waters into sea-ice areas. If temperature seems to be the main shaping factor in water and sea-ice, bacterial communities are important ecological components also in marine sediments, where other variables could affect their phylogenetic composition, i.e., oxygen concentration and nutrient availability. Although knowledge of these sediment communities is considered essential to better understand ecosystem functioning, efforts to study these communities in polar environments have been poor. In Antarctic superficial sediments, Bowmann and McCuaig [109] demonstrated the abundance of *Proteobacteria* (*Gamma*- and *Delta*-), *Flavobacteria* and *Planctomycetales*, and a high representation of groups involved in sulfur cycling and a chemoorganotrophic lifestyle. *Proteobacteria* and *Bacteroidetes* were the most abundant phyla in the Arctic deep-sea sediment, while *Chloroflexi* and *Actinobacteria* were detected in lower numbers. Among *Proteobacteria*, A*lpha*- and G*amma*- were most abundant classes together with *Flavobacteria*, while *Flavobacteriales* and *Rhodobacterales* were dominant at the order level [110]. These findings suggest a sediment bacterial community that differs in composition from that retrieved in polar waters and sea-ice samples. In the following paragraphs and summarized in Table 2, the main physiological and molecular effects/responses observed for bacteria exposed to polar stressing conditions are reported.

### 3.1. Physiological Responses to Environmental Stressors

To cope with polar stress conditions, bacteria have adopted a plethora of protective strategies resulting in morphological and metabolic alterations. Among stressors, low temperature certainly represents the main factor affecting microbial life in cold environments, as it lowers biochemical reaction rates, increases viscosity of solvents and solubility of gasses (e.g., oxygen molecules and reactive oxygen species, ROS), negatively influences solute transport and diffusion, and causes ice formation and osmotic stress, with deleterious effects on biological processes [112,133]. Such transformations do not simply derive from a short-term acclimatization phenomenon, but rather they are driven by permanent and genetically regulated modifications, as discussed in Section 3.2. In general, primary metabolism is downregulated under cold conditions, while a non-classical metabolism becomes activated for a cold lifestyle [111].

A number of vital cell functions (e.g., respiration and transport processes) could be negatively affected by changes in the physical characteristics (i.e., an increase in rigidity) of the cell envelopes, which represent a dynamic interface between the inside and outside of the bacterial cell. The maintenance of membrane fluidity in cold-adapted bacteria is therefore assured by modulating the amounts and types of PUFAs and methyl branched fatty acids (including fatty acid chain length, preferably shorter to establish less interactions, and *cis* to *trans* fatty acids proportions), proteins and carotenoids [91,113,114]. Most cold-adapted bacteria produce *n*−3 poly-unsaturated fatty acid (3-PUFAs) [134,135]. The growth of eicosapentaenoic acid (EPA)-less mutants of *Shewanella livingstonensis* from Antarctic seawater was enhanced at low temperature by the addition of EPA, which is a precursor of PUFA, in the culture medium [136]. Gentile et al. [134] scored the production of PUFAs by *Shewanella* GA-22 (from Antarctic surface seawater) under cultivation with different carbon sources and at different temperatures. A proportion of unsaturated fatty acids up to 70–78% was observed when the strain GA-22 was grown at low temperature. A high prevalence of PUFA-producing bacteria (mainly EPA and docosahexaenoic acid, DHA) was also determined in bacteria isolated from Arctic invertebrates (e.g., copepods, amphipods and bivalves) [137]. Beside maintaining the fluidity of cell membranes, according to Yoshida et al. [138] long-chain PUFAs generate hydrophobic edges between the membrane lipid bilayers, thus shielding the cells from the entrance of ROS (e.g., H_2_O_2_) into the cells. Therefore, their presence in cold-adapted bacteria may be a response to oxidative stress, which is enhanced at low temperatures.

Membrane fluidity is also improved by non-polar carotenoids, associated with cell membranes and acting as fluidity modulators under cold conditions [112]. This was demonstrated, for example, for *Sphingobacterium antarcticus* and *Micrococcus roseus* from Antarctica [139,140]. Moreover, an increased resistance to environmental stressors such as freeze-thaw cycles and exposure to solar radiation was observed in carotenoid-pigmented bacteria rather than in non-pigmented strains, suggesting that both photo- (e.g., against intense light range and UV irradiation) and cryoprotection roles are played by carotenoids in polar environments [141]. More recently, Singh et al. [142] investigated the effect of freeze-thaw cycles and temperature on pigmented bacterial strains (i.e., *Leeuwenhoekiella aequorea*, *Pseudomonas pelagia*, *Halomonas boliviensis*, *Rhodococcus yunnanensis*, and *Algoriphagus ratkwoskyi*) isolated from the Arctic Kongsfjorden. The concentration of zeaxanthin-like pigments was found to be 26–65% higher after freeze–thaw, suggesting that they probably confer better cryoprotection by regulating the fluidity of membranes.

Among other cell envelope components, peptidoglycan thickening in Gram-positive bacteria also contributes to their protection against freezing-thawing pressure, cell disruption deriving from ice formation, and osmotic imbalance at low temperatures [115,116]. For example, *Planococcus halocryophilus* Or1 adopts a peculiar mechanism to thicken the outer cell surface by displaying hydrophobic encrustations composed of peptidoglycan, calcium carbonate and choline [115]. According to Corsaro et al. [117], lipopolysaccharides (LPS; constitutive components of the outer membrane in Gram-negative bacteria) lack the O-chain component, and are thus shorter in length. This alteration seems to increase the outer membrane flexibility and stability [117]. Similarly to lipids in the cell membrane bilayer, the lipid A component of LPS shows a higher content of short chain and/or unsaturated fatty acids, thus contributing in an enhanced membrane fluidity [117,118].

To overcome the main adverse effect of low temperature, i.e., the reduction of cellular reaction rates (including some vital processes, such as transcription and translation), cold-adapted bacteria produce enzymes with high catalytic activities, which may be 10 times higher at low temperatures than those of their mesophilic counterparts under cold conditions [91,119]. An appropriate rate for enzyme-catalyzed reactions is gained thanks to an increased structural flexibility or plasticity of specific regions (e.g., near or at the catalytic site) or of the whole protein. Structural adjustments include: The reduction in number of residues (i.e., proline and arginine that by restricting backbone rotations and forming multiple hydrogen bonds and salt bridges, respectively, stabilize the protein), especially in loops bordering the active site; the replacement of bulky side chains with smaller groups at the entrance to the active site (e.g., glycine that essentially has no side chains, thus allowing a localized chain mobility) and a greater exposure of hydrophobic residues [116,143]. All these modifications allow the enzyme, and the catalytic center (both the release and exit of the reaction products are improved), to be more flexible at low temperatures that could instead reduce molecular motion by a freezing action. Thus, structural adaptation strategies, which however differ among enzymes, result in a less compact 3D structure that enables enzymatic activity at a low energy cost, even if this leads to a reduced stability and a higher thermolability (as a consequence, highly active enzymes at low temperature are preferred to stable proteins).

Bacterial survival under extremely low temperature conditions is also enhanced by proteins with peculiar properties and functions. Among these, antifreeze (AFPs) and ice nucleating (INPs) proteins play key roles in cold adaptation. AFPs are able to bind to ice crystals, inhibiting their growth and ice recrystallization [120,121]. Thanks to the action of AFP the freezing temperature of a solution containing ice is lowered below the melting point of the ice (a process known as thermal hysteresis). According to Raymond et al. [144], AFPs are involved in the stabilization of brine pockets for the preservation of a liquid environment in the cell proximity. In addition to freeze avoidance and freeze tolerance, AFPs seem to possess a third envirotactic role, i.e., transient adhesion of an organism to ice, thus gaining access to oxygen and nutrients which are potentially more abundant in the upper water column, as observed for the Antarctic bacterium *Marinomonas primoryensis* from a brackish lake [121]. INPs are membrane-bound proteins that enhance the formation of ice crystals at temperatures close to melting point, stabilizing water molecules in an ice-like structure and inhibiting intracellular ice formation, thus avoiding cryoinjury of the cells [145].

In addition to AFPs and INPs, to contrast the deleterious, and often lethal, effects of sub-zero temperatures leading to the formation of intracellular (with the subsequent cryoinjury) or extracellular (causing high concentrations of extracellular solutes and provoking osmotic stress, as well as removal of liquid water for vital biological functions) ice crystals, cold-adapted bacteria produce a variety of efficient cryoprotective compounds, including compatible solutes, extracellular polymeric substances (EPSs) and polyhydroxyalkanoates (PHAs). Compatible solutes (such as glycine sucrose, mannitol, sorbitol, trehalose, and betaine) are low-molecular mass organic osmolytes that, being accumulated in the cytoplasm by cold-adapted bacteria, lower the cytoplasmic freezing point and avoid desiccation by counteracting water loss and cell shrinkage during freezing [116]. EPSs are high molecular weight biopolymers consisting mainly of homo- or hetero-polysaccharides, but they may also have organic and inorganic substituents in their structure (e.g., proteins, nucleic acids, lipids, and humic substances). EPSs can be covalently (i.e., capsular polysaccharides forming a protective shell around the cells) or loosely (i.e., slime polysaccharides, released into the environment) bound to the cell surface [146]. A plethora of ecological roles are played by EPSs in cold environments. Beside acting as osmoprotectants, EPSs produced by cold-adapted bacteria have been reported as ROS scavengers and cryoprotectants [147,148,149,150]. For example, high concentrations of EPSs are produced at low and sub-freezing temperatures by *Pseudoalteromonas*, *Winogradskyella* and *Marinobacter* isolates, probably acting as a diffusion barrier to solutes and a physical-like barrier to ice formation [148,149].

Finally, PHAs are microbial polyesters synthetized by a number of bacteria and accumulated in the cytoplasm in response to nutritional limitation or stressful conditions. They constitute a dynamic reserve of carbon, nitrogen, reducing equivalents and energy at the cellular level [111]. Beside their involvement in cryoprotection, PHAs act also in processes such as oxidative stress resistance, maintenance of cellular redox balance and cell motility. As an example, polyhydroxybutyrate (PHB) accumulated by *Pseudomonas extremaustralis* affect biofilm formation and motility under cold conditions [111].

### 3.2. -Omics Studies on Bacteria from Cold Environments

The advent and development of new -omics technologies have shed light on the cold lifestyle mechanisms of bacteria, also allowing access to information on the non-cultivable fraction. To date, over 100 genomes of cold-adapted microorganisms (Bacteria and Archaea) are available and have provided interesting insights on their unique capabilities [151]. The general agreement of many studies claims a complex genetic regulation system, in which they adapt themselves to the cold environment thanks to specific genetic traits and limiting or silencing the expression of unnecessary genetic traits. As pointed out by Bowman [151], in front of a strong effort in bacterial genome sequencing during recent years, very few data are proportionally available on strictly cold-adapted bacteria.

Studies at the genome-level have been performed on several psychrophilic or psychrotolerant bacterial genera, such as *Shewanella*, *Octadecabacter*, *Glaciecola*, *Psychroflexus*, *Paenibacillus* [122,126,152,153] evidencing common and different features of psychrophily. Different gene traits correlated to usual mechanisms of response to stress factors have been found in different cold-adapted bacteria. Generally, common genetic traits include those related to oxidative stress, metabolism and energy and nutrient acquisition, cell wall membrane structure and fatty acid biosynthesis, production of cold-shock protein (CSP) and chaperones, production of exopolysaccharides or other extracellular substances. Biosynthesis or transport of compatible solutes (i.e., glycine betaine, ectoine, and trehalose), are also used by cold-adapted bacteria to regulate turgor pressure or to increase molecular and membrane stability [127].

The presence of genes involved in antioxidant activity, such as superoxide dismutase, glutathione peroxidase, glutathione reductase, catalase, aconitase, thioredoxin and ascorbic acid were identified in the genome of *Colwellia* sp. Arc7-D, a H_2_O_2_-resistant psychrophilic bacterium, isolated from Arctic Ocean sediment [123]. Additionally, a glycogen operon consisting of phosphoglucomutase, glucose-1-phosphate adenylyltransferase and quinoprotein glucose dehydrogenase was detected. Glycogen synthesis is a known mechanism used in the cold lifestyle to prolong survival during growth-limiting conditions [124]. A similar study performed on *Colwellia psychrerythraea* 34H confirmed important capabilities of such genus in carbon and nutrient cycling, bioremediation, production of secondary metabolites, and cold-adapted enzymes [154]. Likewise, genome annotation performed on the sea-ice bacterium *Psychromonas ingrahamii* 37 by Riley et al. [155] showed a large number of regulators of cyclic GDP (possibly involved in the production of extracellular polysaccharides), genes for the production of several agents acting to balance osmotic pressure (i.e., osmolyte, betaine choline) and three-subunit TRAP systems (Tripartite ATP-independent periplasmic transporters) involved in nutrient transport at low temperature. Moreover, the authors evidenced some groups of gene products possibly correlated to proteins with unknown functions implied in psychrophily and emphasized the importance of proteomic studies to better address this issue.

Recently, the genome annotation of the novel Arctic strain species *Flavobacterium cellulosilyticum* AR-3-4^T^ [156] revealed the presence of genes involved in the adaptation to low temperatures, and the presence of cold shock domain-containing proteins related to cold adaptation. In addition, the presence of a gene cluster encoding for glycogen synthase and 4-oxoacyl-ACP reductase, involved in the ability to survive in cold regions [125], was also detected, as well as the putative secondary metabolite biosynthesis gene clusters for terpene, nonribosomal peptide synthetase (NRPS), and different polyketide synthases (T1PKS and T3PKS).

The most promising approach to identify the different adaptations from the genomic point of view is the comparison between strains belonging to the same taxonomic group, but subjected to different environmental pressures. In this context, other genomic-level studies were focused on the comparative analysis between psychrophiles and mesophiles and/or thermophiles bacteria and were aimed at detecting differences which could explain the adaptability level according to the respective ecological niches. As an example, Feng et al. [122] compared the genomes of the psychrophilic bacterium *Psychroflexus torquis* ATCC 700755T and its closely affiliated non psychrophilic *P. gondwanensis* ACAM 44T, demonstrating more pronounced horizontal gene transfer phenomena in the cold-adapted strain. The highly translated genes in *P. torquis* were related to exopolysaccharide and polyunsaturated fatty acid biosynthesis, or involved in nutrient acquisition, production of proteins associated with ice-binding and light-sensing processes that were mostly absent in *P. gondwanensis* strain. High levels of horizontal gene transfer phenomena were demonstrated also in polar *Octadecabacter* members, namely *O. arcticus* 238 and *O. antarcticus* 307, isolated from Arctic and Antarctic sea-ice, respectively [152]. The authors demonstrated a high level of genome plasticity and numerous genomic rearrangements in the genome alignments, suggesting a shared distinct gene pool (mainly based on notable conserved gene clusters involved in xanthorhodopsin synthesis, mercury resistance, flagella arrangement and gas vesicle formation) between the strains, which was differently expressed or absent in mesophilic *Roseobacter* clade members. Interestingly, 2% total genome content sharing emerged from the comparison between the two *Octadecabacter* strains, suggesting specific adaptations to the habitats. However, despite low sharing levels, strains were defined as phylogenetically closely related, and distinct from the *Roseobacter* pangenome. Similarly, Zhao et al. [126] compared the genomes of different *Shewanella* members differing in temperature and Na^+^ requirements, and hexahydro-1,3,5-trinitro-1,3,5-triazine degradation capability. Genomic and proteomic analysis evidenced among cold life-style adaptations of *S. halifaxensis* and *S. sediminis* a reduced G+C content and peculiar composition for certain aminoacids to increase protein flexibility.

Despite extremely interesting and fundamental for the total comprehension of the cold lifestyle, genomic studies could gain information essentially on the presence of specific gene groups and predict their potential. The studies conducted so far have highlighted the need to use new -omics in a complementary approach to have a comprehensive overview. If genomic analyses have in fact highlighted the presence of genetic traits which, properly expressed or silenced, favor the survival of bacteria in extreme cold environments, these data need the contribution of the transcriptomic approach to define which of these potentials are exploited by individual strains. The numerous data suggest that psychrophily is not always linked to the presence of exclusive genetic traits, but rather to an important modulation in their expression, or to gene transfer phenomena that provide the single strain the acquisition of specific clusters (i.e., loss of traits that allow growth at high temperatures [151].

Several transcriptomes have been reported for both eury- and steno-psychrophiles from different cold environments. Some common features have been evidenced by Raymond-Bouchard and Whyte [127], mainly consisting in increased transcripts involved in oxidative stress, CSP and chaperone production, metabolism and energy management, membrane fluidity assessment.

Many studies have focused on the different transcriptional activity under different temperature conditions. As an example, a differential expression of CSP and chaperones occurs at different temperatures, but it is not necessarily up-regulated in the case of lower temperature, and they can be expressed in a constitutive mode in particular conditions. This is the case of *P. arcticus*, whose CSPs were constitutively expressed across all temperatures, suggesting a strong predisposition of these microorganisms to react promptly to changes in temperature conditions [131]. Interestingly, a correlation between cold and oxidative stress was found, probably influenced by increased gas solubility and higher enzyme activity at lower temperatures [114]. The cold-induced expression of genes encoding for antioxidant enzymes was detected in *Psychrobacter*, *Planococcus*, *Nesterenkonia* species [115,129,131]. In *Pseudomonas extremaustralis* a repression of genes involved in oxidative stress at colder temperatures and an up-regulation of iron-related proteins was observed, indicating a possible role of these molecules in the oxidative stress response [128]. Moreover, an up-regulation of genes involved in ethanol oxidation (*exaA*, *exaB* and *ExaC*) encoding for a pyrroloquinoline quinone (PQQ)-dependent ethanol dehydrogenase, a cytochrome c550 and an aldehyde dehydrogenase were detected, suggesting that the ethanol oxidation pathway could be implied in energy production at low temperatures, even in the absence of exogenous ethanol [128].

The transcriptomic and proteomic analyses performed for *Exiguobacterium antarcticum* B7 grown at 0 °C and 37 °C revealed that four out of six CSPs were expressed at low temperature, and a higher abundance of CSPs was evidenced in the proteome at 0 °C. A differential expression of genes at the two growth temperatures was detected, indicating a probable relation to the maintenance of transcription and translation processes and to macromolecules integrity, as a strategy to overcome the low temperature [157]. The polyextremophile strain *Nesterenkonia* sp. AN1 isolated from Antarctic desert soil possesses a core genome encoding for several proteins involved in universal and cold stress response, in contrast to other mesophilic *Nesterenkonia* spp. strains. An induction of transcripts encoding for antioxidants (suggested by the up-regulation of *sodA*, *bcp* and *bpoA2*), and of genes encoding for key enzymes of the glioxylate cycle (isocitrate lyase and malate synthase) were revealed during growth at 5 °C, suggesting a possible adaptive strategy to cold habitats [129]. An interesting experiment of temperature downshift from 30 °C to 8 or 15 °C was applied to *Shewanella oneidensis* MR-1 to check the transcriptional response. Interestingly, more than 700 genes were significantly affected by the cold shock, evidencing a remarkable differential expression of genes, *So1648* and *So2787* involved in the cold shock response. Expression for several protein families (i.e., membrane and regulatory proteins, metabolic proteins, especially those involved in NADH and NADPH generation), DNA metabolism and translation apparatus components resulted up-regulated [130].

The transcriptomes obtained from bacteria growing below zero have been less explored. Bergholz et al. [131] studied *Psychrobacter arcticus* 273-4 from permafrost environment and analyzed the transcriptome during growth at different temperatures, from −6 °C to 22 °C. A fast-growth state and a resource efficiency state were detected for the strain at higher (17 °C) and lower temperature (below 4 °C), respectively. At low temperature, a down-regulation of genes involved in transcription, translation, energy production, and most biosynthetic pathways were reported, while genes for specific biosynthesis processes (proline, tryptophan, and methionine), RNases and peptidases were generally up-regulated. Differently from other psychrophiles and mesophiles, *clpB* and *hsp33* encoding for chaperones were up-regulated at low temperature. Slightly different results derive from a similar study performed more recently by Koh et al. [132] on the permafrost soil *Psychrobacter* strain PAMC 21119. The transcriptomic and proteomic analysis performed at −5 °C and 20 °C revealed an up-regulation of genes for translation, ribosomal structure and biogenesis and a down-regulation of lipid transport and metabolism at low temperature. At the proteomic level, up-regulation was detected for proteins involved in metabolite transport, protein folding and membrane fluidity, while those related to energy production and conversion were down-regulated. Protein expression of *MetF*, *ScoB* and *MmsA,* involved in aminoacid biosynthesis were induced at low temperature, but the tricarboxylix acid (TCA) cycle was not activated at −5 °C, thus suggesting that the acetyl-CoA input into TCA cycle is shunted to glutamine synthesis. Interestingly, the function of nine protein spots, with proved differential expressions, were not assigned, as also found for other transcripts with unknown functions in *Exiguobacterium sibiricum* and *Planococcus halocryophilus* Or1 [115,158]. Another interesting aspect that emerges from the study of Koh and coauthors [132] is the discrepancy between the transcriptome and proteome, partially justified from the numerous processes occurring between transcription and translation to protein as end-point, but still worthy of further investigations. An example of multi-omics approach (an integration of genomic and phenomic) was applied for the strain *Pseudoalteromonas haloplanktis* TAC125 (PhTAC125) [2], in the attempt of merging genome and phenotypic features. By comparing the metabolic assessment of the strain with the related data available on the Antarctic bacterium *Pseudoalteromonas* sp. TB41 (PspTB41), the study underlined a higher metabolic versatility of *P. haloplanktis* TAC125 at 4 °C, as suggested by its capacity to enhance the uptake of compounds with a peculiar role in cryoprotection (i.e., spermine, glutathione, ornithine and other compounds related to glutathione metabolism). A stronger enrichment of PhTAC125 genome in genes involved in lipid transport and metabolism and an up-regulation of protein synthesis metabolism at low temperatures were also detected, in addition to its remarkable efficiency in the use of sulfur, phosphorous and N compounds, denoting an enhanced ability to produce aminoacids and proteins under cold conditions. Although the mutagenic approach was not considered in this review, it is worth pointing out that the role of genes encoding for enzymes involved in tRNA modification (as tRNA-modifying enzyme TrmE) and codifying for GTPase [159] and aspartate aminotransferase [160] in cold adaptation was demonstrated in mutant strains. Moreover, cyanobacteria have been particularly considered as models for the study of membrane fluidity adaptations, through genetic manipulation of desaturase genes [161,162]. It has been demonstrated that the expression of three desaturases (encoded by *desA*, *desB*, and *desD*) is inducible at cold temperature in the cyanobacterium genus *Synechocystis.* In conclusion, as also suggested by Methè et al. [154], the -omics analyses provide a picture in which cold adaptation is not represented by the presence of unique gene sets, but from a collection of genome changes in terms of gene content and aminoacid composition. Therefore, in general we can assume that cold-adapted bacteria show the tendency to reduce energy consumption to reserve it while waiting for better environmental conditions, as a form of hibernation. Moreover, different approaches could provide different conclusions and perspectives.

## 4. Phyto-Bacterioplankton Interactions in Cold Environments

In the Antarctic sea-ice, microalgae and bacteria coexist and cope with harsh environmental conditions by secreting extracellular polymers and ice-active substances, or by modulating their own metabolism to suit their partner’s needs [163]. To date, the relationships between these two components within microbial communities have been scarcely investigated. Both groups are very sensitive to external environmental factors and their coexistence, distribution and interactions can be a key for understanding the ecological dynamics affecting polar areas. This section aims at exploring the studies carried out so far on the topic and at drafting a baseline for future research. Most studies linking the distribution and diversity of bacteria and microalgae in cold environments have been focused on certain phytoplankton classes, mainly diatoms and phytoflagellates. Several researchers have tried to better understand these interactions by correlating bacterial distribution with the high chlorophyll levels in the Antarctic sea ice, and proposed sea-ice diatom assemblages as habitats for the establishment of psychrophilic and psychrotrophic bacterial communities [164,165].

In some cases, the study of interactions between algal and bacterial components is addressed to elucidate possible succession in taxonomic groups within bacterial communities in relation to phytoplankton blooms. This is the case of the recent study by Rapp et al. [166], who reported an intriguing analysis of the community composition, turnover and interaction of prokaryotic and eukaryotic microorganisms with ice-associated and sinking algal aggregates. The phytoplankton community was dominated by diatoms, dinoflagellates and other alveolates, with richness and diversity peaks in the sea-ice habitats. The authors concluded that the algal deposit at the seafloor possesses a unique community structure, with a 22% and 17% operational taxonomic unit (out) overlap with the original sea-ice community and with the deep-sea community, respectively. Bacterial communities were differently represented in the different matrices, dominated by *Flavobacteria*, *Gamma*- and *Alphaproteobacteria* (*Flavobacterium*, *Polaribacter*, *Psychroflexus*, *Nonlabens*, *Colwellia*, *Glaciecola*, *Planktomarina* most abundant genera) in ice and water samples, whilst a predominance of *Deltaproteobacteria*, *Acidimicrobia* and Verrucomicrobiae members occurred in deep-sea surface sediments. The authors found that algal aggregates acted a specific selection for flavobacterial affiliates and gammaproteobacterial *Glaciecola* and *Paraglaciecola*, all known for their strong relation with phytoplankton bloom events [167] and the ability to hydrolyze vegetable polysaccharides, denoting also a succession of these bacterial taxa in dependence on the availability of primary production products.

Even if some correlations between bacterial and phytoplankton populations have been reported in cold ecosystems, they have been attributed mostly to the turnover of nutrients and organic matter, thus mainly indicating the role of such interactions in the context of ecological dynamics. Several studies argued about the intimate and selective character of interactions between phytoplankton and bacteria, by indicating that specific bacteria are associated with most phytoplankton cultures and algal blooms [168,169,170,171], supporting the existence of phytoplankton-associated bacterial taxa [172]. In this context, the term “phytosphere resurfaces” has been suggested to indicate the area surrounding an algal cell or colony in which bacterial growth is enhanced by algal extracellular products [173]. Seymour et al. [17] described the phytosphere as a sort of ecological oasis for heterotrophic bacteria, in which they can have access to high concentrations of organic carbon. The occurrence of specific associations between phytoplankton and bacteria in cold environment was also assessed by Zhen et al. [174], who investigated the bacterial community in four marine Arctic microalgae, namely *Micromonas* sp., *Fragilariopsis* sp., *Attheya septentrionalis* and *Thalassiosira* sp. and glacial *Chlorella* sp. The study described the coexistence of phytoplankton and bacteria in the Arctic ecosystem, by showing stability in the phycosphere bacterial community structure dominated by the cyanobacteria, *Alpha*- and *Betaproteobacteria* in marine microalgae, whilst CFB (*Cytophaga*-*Flexibacter*-*Bacteroides*) group was detected only in the phycosphere of *Micromonas* sp., *Fragilariopsis* sp., and *Chlorella* sp.

Indications of relationships more strictly configured as symbiosis between such microbial organisms are still scarce and inconsistent and need specific cultivable approaches. Although in situ observations suggest a possible specificity, some results from batch culture studies do not seem to support this specificity. An interesting attempt to address this issue was focused on the bacterial communities associated with seven diatom and dinoflagellate species isolated from Helgoland Roads, North Sea [175]. The authors studied the bacterial diversity and succession in batch cultures, for a period of 8 weeks, by monitoring bacterial abundance, changes in algal photosynthetic efficiency and consumption of inorganic nutrients over time. *Alpha*- and *Gammaproteobacteria*, together with *Flavobacteria*–*Sphingobacteria* group members, resulted predominant in all cultures, with differences occurring in free-living and attached bacterial populations, but without evidence of correlation with algal growth, thus suggesting that a specificity in the associations of bacterial taxonomic groups and algal species was not detected. Conversely, only in the case of the alga *Guinardia delicatula*, a strong succession in the bacterial community and a clear separation between free-living and attached bacteria was highlighted during growth. All microalgal species shared most of the associated bacterial groups, with the only exception of the diatom *Thalassiosira rotula*, who harbored a specific bacterial community. This should support the already assumed theory of a strict correlation [176,177]. Notwithstanding, it should be pointed out that the same microalgal species collected in different seasons (spring and summer) could act as hosts of different bacterial populations. Despite the conflicting opinions, the possible specificity could depend on reasons linked to the single algal taxonomic group, or on the difficulty to reproduce the actual environmental conditions in the laboratory, especially in the case of cold-adapted species.

Delmont et al. [178] studied the interaction of the bacterial community with the algal *Phaeocystis antarctica* bloom in the Amundsen Sea polynya, highlighting the coexistence with the bacterial taxa *Polaribacter sensulato*, acryptic *Oceanospirillum*, SAR92 and *Pelagibacter*. A strong influence of the *Phaeocystis* bloom in shaping heterotrophic bacterial communities was suggested, as supported by the involvement of bacterial populations in the evolution of the algal bloom, starting from its formation, with a high incidence of some taxa (especially SAR92), until its decline when a high incidence of *Colwellia* and Cryomorphaceae members. This influence was not only related to the potential consumption of algal exudates by bacteria. *Colwellia* members are known for their role in nutrient cycling through degradation of bloom biomasses [179], and for the production of extracellular products and enzymes [149] with a specific role in the degradation of high molecular weight compounds [154]. Interestingly, this shows how different factors are involved in the relationships between algae and bacteria in cold seas, such as the ability to degrade organic substances of algal derivation, and involvement as both free-living and epibiontic bacteria. Several hypotheses have attempted to explain the occurrence of bacteria associated with sea-ice. Nutrient accessibility by algal exudates, possibility to colonize the algal surface, the nutritional relationships based on the turnover of dead organic material, or the possible role of bacteria in defending the diatoms from reactive oxygen species have been speculated [180]. Hünken et al. [181] tried to understand the interactions between the Antarctic ice diatom *Amphiprora kufferathii* and its epiphytic bacterial fraction. The study performed on axenic and non-axenic cultures of *Amphiprora kufferathii* indicated a decreased density of diatoms in culture without epiphytic bacteria, but the mutual benefits deriving from bacterial remobilization of nutrients and the algal providing of carbon source for their symbionts was not enough to explain the nature of the relationship. Interestingly, the authors found a bacterial consumption of H_2_O_2_ from the medium, with a reduction 30% faster than in axenic cultures, and with beneficial effects also in stressful light conditions for the diatoms, suggesting a significant contribution to the diatom’s antioxidant defences and the enhancement of their growth in special environmental systems, such as brine cavities in Antarctic sea-ice. The bacterial strains isolated from cultures resulted affiliated with *Bacteroidetes* (*Pibocella* spp.), *Alpha*- (*Sulfitobacter* spp.) and *Gammaproteobacteria* (*Colwellia* spp.) and were equipped with specific enzymes involved in oxidative chemical reactions.

To date there have been few omics approaches to study this issue, but some attempts have demonstrated that the support of innovative approaches could allow to go deeper, exploiting gene expression as a key to understand the dynamics of phytoplankton-bacterial interactions. This was shown, for example, in the bacterial management of iron and cobalamin in co-limitation conditions [182]. The authors investigated changes occurring in global gene expression induced by shifts in micronutrient availability. Expression of the cobalamin biosynthetic pathway was detected in the gammaproteobacterial population of *Oceanospirillaceae* ASP10-02a. A mutualistic relationship was suggested by the concomitant expression of genes involved in organic matter acquisition and cell surface attachment, indicating its dependence on phytoplankton growth to influence cobalamin production. In addition, a competitive relationship between bacterial *Methylophaga* groups and phytoplankton, as cobalamin consumers, was determined.

## 5. Conclusions

**Figure 2:**
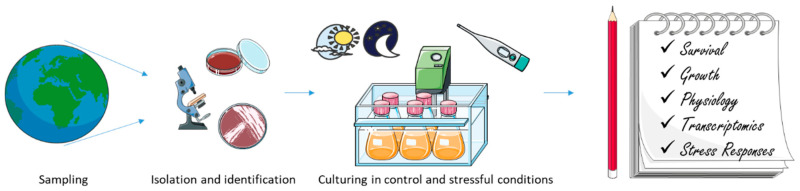
The figure summarizes the most common pipeline used in order to study microbial responses to main environmental stressors (e.g., low temperature and high light).

Polar microbial communities are a critical component of polar marine ecosystems, contributing 10–50% of the annual primary production of polar seas, supporting overwintering zooplankton species, especially Antarctic krill, and seeding spring phytoplankton blooms. Several studies have been performed on polar microorganisms in order to understand their response to environmental stressors (on samples collected in the natural environment or polar species cultivated in laboratory conditions) and to predict the possible trends in a changing environment (Figure 2). Climate change in polar regions is expected to be among the largest and most rapid of any region on the Earth [183,184].

**Figure 3:**
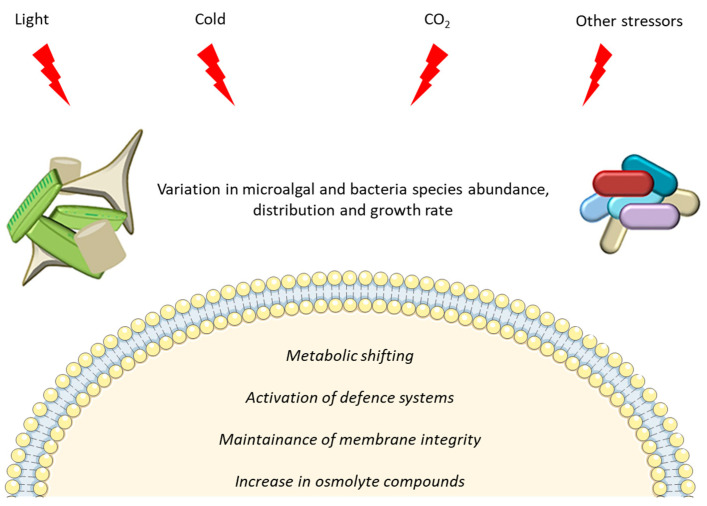
Representation of main effects induced in microalgae and bacteria by polar environmental stressors.

The role played by microalgae and bacteria in marine environments is crucial, and becomes even more pivotal in extreme cold environments, where external conditions require peculiar adaptations and ecosystem balances. They live in environments with multiple abiotic factors that change simultaneously, act in combination or are inter-dependent. Despite their inhospitality, polar ecosystems host a great biodiversity, with diverse and active communities of microorganisms. Symbiotic relationships allow precious opportunities of survival to bacteria and microalgae, more than those determined by mutations or competition [185]. It may be considered as one of the key mechanisms in future adaptation strategies to cope and overcome unprecedented environmental modifications due to climate change and anthropogenic pressure [186,187]. Altogether, the data reported in this manuscript show that increase in light exposure, exposure to cold, increase in CO_2_ concentration and/or combination of stressors induce differences in growth rate, and species abundance and distribution for both polar bacteria and microalgae. At the cellular level, a metabolic shift is observed increasing the expression of transcripts related to lipid metabolism and defence systems (such as the up-regulation of IBP, chaperones and antioxidant enzymes) (Figure 3).

This review highlights that -omics resources for polar species are still few. In addition, the available studies have identified, both for bacteria and microalgae, several sequences with still unknown functions. Hence the need to further study polar environments, to deeply understand the biology and ecology of the inhabiting species, such as bacteria and microalgae, and their interactions, for which many aspects are still unexplored but highly promising.

## Figures and Tables

**Table 1:** Main physiological and molecular effects/responses observed for microalgae exposed to stressful conditions encountered in polar environments.

Stress Exposure	Main Effects/Responses	Reference
Physiological changes		
Low salt concentration	Increase in specific growth rate, biochemical composition shifting towards a lower POC:PON relationship with higher protein content, but reduced fatty acid and carbohydrate content.	[27,44]
High salt concentration	Slowing down of cell division and growth rate, reduced cell size and motility, triggering “palmelloid” formation in *Chlamydomonas,* production of osmoregulatory compounds (e.g., glycerol and proline), increase in ion transmembrane transport and lipids. In the sea-ice diatom, *Nitzschia lecointei*, small changes in growth rate, effect on cellular metabolite pool sizes	[46,47,48]
High levels of ultraviolet radiation (UVR)	Increase in photoprotective pigments	[49,50]
	Reduction in photosynthetic rate	[51]
	Inactivation of specific enzymes	
	affecting species diversity and richness.	
	Increase in saturated fatty acids, decrease in polyunsaturated fatty acids (PUFAs), small increase in C18 PUFAs	[52]
Low light exposure	Protection from UVR	
	Limitation in primary production	
Nutrients: Fe limitation	Influence in microalgal growth and composition	[53]
	Increase in lipid production	[54]
Low pH	Increase in large diatoms, early senescence	[55]
	Increase in photosynthetic rate and growth rate	
	Affecting membrane potential, energy partitioning and enzyme activity	[56]
Low temperature	Maintenance of membrane fluidity thanks to unsaturated fatty acids	[57]
	Maintenance of sufficient rates of enzyme-catalyzed reactions for key metabolic processes	
	Evolution of cold shock and antifreeze proteins	
	Photosynthetic electron transport chain adaptations	
Molecular changes		
Genome comparison between cold-adapted and temperate species	In the cold-adapted genome there were highly divergent alleles which were also differentially expressed across various environmental conditions. Main genes identified for cold adaptation were ice-binding proteins IBPs, proton-pumping proteorhodopsins and chlorophyll a/c light-harvesting complex LHC.	[58]
Low temperature, high light	Genes encoding proteins of PSII (*psbA*, *psbC*) and for carbon fixation (*rbcL*) were down-regulated	[59]
	Chaperones (*hsp70*) and genes for plastid protein synthesis and turnover (elongation factor *EfTs*, ribosomal protein *rpS4*, *ftsH* protease) were up-regulated	
Low temperature, low light	Down-regulation of *psbA*, *psbC*, and *rbcL*	[59]
Low temperature, high salinity	Ionic transporters and antiporters, heat shock proteins, genes related to oxidative stress, and three key genes involved in proline synthesis	[60]
Low temperature	Expression of various DEAD-box RNA helicase genes, such as *CiRH5*, *CiRH25*, *CiRH28*, and *CiRH55*, were found significantly up-regulated under freezing treatment	[61]
	Increase in genes encoding proteins involved in protein translation and transport, including protein transport protein SEC61, signal recognition particle protein, protein involved in vacuolar protein sorting. Increase in Heat shock protein 70, matrix metalloproteinase M11, X-Pro dipeptidyl-peptidase, and protein binding 26S proteasome regulatory complex, nitrate reductase, ferredoxin-nitrite reductase, and nitrate/nitrite transporter.	[62]
Increased temperature	Up-regulation of cytoprotective genes, down-regulation of genes related to photosynthesis, increase in fucoxanthin chlorophyll a/c-binding proteins	[63]
High light	Transcripts related to photosynthesis were affected	[64]
	Identification of an antifreeze protein gene (*Cn-AFP*)	[65]
High salinity	Increased expression of genes participating in the metabolism of carbohydrates, such as starch, sucrose, soluble sugar, and glucose	[66]

**Table 2:** Main physiological and molecular effects/responses observed for bacteria exposed to the stressful conditions they could encounter in polar environments.

Stress Exposure	Main Effects/Responses	Reference
Physiological changes		
Low temperature	Reduction of primary metabolism	[111]
	Modulation of PUFA amount for membrane fluidity maintenance, modulation of proteins and carotenoids	[91,112,113,114]
	Peptidoglycan thickening	[115,116]
	LPS lacking O-chain component	[117]
	Increase in short chain and/or unsaturated fatty acids	[117,118]
	Increase in enzymes with high catalytic activity	[91,119]
	Increase in antifreeze (AFPs) and ice nucleating (INPs) proteins	[120,121]
	Increase in cryoprotective compounds, including compatible solutes, extracellular polymeric substances (EPSs) and polyhydroxyalkanoates (PHAs)	[116]
Molecular changes		
Genomic observations	Common genetic traits include those related to oxidative stress, metabolism and energy and nutrient acquisition, cell wall membrane structure and fatty acid biosynthesis, production of cold-shock protein (CSP) and chaperones, production of exopolysaccharides or other extracellular substances, biosynthesis or transport of compatible solutes (i.e., glycine betaine, ectoine, and trehalose), and presence of genes involved in antioxidant activity, such as superoxide dismutase, glutathione peroxidase, glutathione reductase, catalase, aconitase, thioredoxin and ascorbic acid	[122,123]
	Glycogen synthesis genes	[124]
	Presence of gene cluster encoding for glycogen synthase and 4-oxoacyl-ACP reductase, putative secondary metabolite biosynthesis gene clusters for terpene, nonribosomal peptide synthetase (NRPS), and different polyketide synthases (T1PKS and T3PKS)	[125]
	Presence of genes related to exopolysaccharide and polyunsaturated fatty acid biosynthesis, or involved in nutrient acquisition, production of proteins associated with ice-binding and light-sensing processes	[122]
	Reduced G+C content and peculiar composition for certain aminoacids to increase protein flexibility	[126]
Transcriptome analyses at low temperature	Up-regulation of transcripts involved in oxidative stress, CSP and chaperone production, metabolism and energy management, membrane fluidity assessment	[127]
	Up-regulation of genes involved in ethanol oxidation (*exaA*, *exaB* and *ExaC*) encoding for a pyrroloquinoline quinone (PQQ)-dependent ethanol dehydrogenase, a cytochrome c550 and an aldehyde dehydrogenase	[128]
	Induction of transcripts encoding for antioxidants (suggested by the up-regulation of *sodA*, *bcp* and *bpoA2*), and of genes encoding for key enzymes of the glioxylate cycle (isocitrate lyase and malate synthase)	[129]
	Expression for several protein families (i.e., membrane and regulatory proteins, metabolic proteins, especially those involved in NADH and NADPH generation), DNA metabolism and translation apparatus components resulted up-regulated	[130]
	Down-regulation of genes involved in transcription, translation, energy production, and most biosynthetic pathways was evidenced, while genes for specific biosynthesis processes (proline, tryptophan, and methionine), chaperones *clpB* and *hsp33*, RNases and peptidases were generally up-regulated	[131]
	Up-regulation of genes for translation, ribosomal structure and biogenesis and a down-regulation of lipid transport and metabolism	[132]
Proteome analysis at low temperature	Up-regulation was detected for proteins involved in metabolite transport, protein folding, membrane fluidity and aminoacid biosynthesis (protein MetF, ScoB and MmsA). Proteins related to energy production and conversion were down-regulated	[132]
Multi-omics (Genomic and Phenomic) analysis	High metabolic versatility, capacity to enhance the uptake of compounds with peculiar role in cryoprotection (i.e., spermine, glutathione, ornithine and other compounds related to glutathione metabolism). Enrichment in genes involved in lipid transport and metabolism and an up-regulation of protein synthesis metabolism	[2]
